# Color Design Decisions for Ceramic Products Based on Quantification of Perceptual Characteristics

**DOI:** 10.3390/s22145415

**Published:** 2022-07-20

**Authors:** Yi Wang, Qinxin Zhao, Jian Chen, Weiwei Wang, Suihuai Yu, Xiaoyan Yang

**Affiliations:** 1 School of Design and Art, Shaanxi University of Science and Technology, Xi’an 710021, China; 201022011@sust.edu.cn (Q.Z.); chenjian@sust.edu.cn (J.C.); wangweiwei@sust.edu.cn (W.W.); caid@nwpu.edu.cn (S.Y.); yangxiaoyan@sust.edu.cn (X.Y.); 2 School of Mechanical Engineering, Northwestern Polytechnical University, Xi’an 710051, China

**Keywords:** industrial design, perceptual semantics, ceramic color, feature quantification, neural network

## Abstract

The appearance characteristics of ceramic color are an important factor in determining the user’s aesthetic perception of the product. Given the problem that ceramic color varies and the user’s visual sensory evaluation of color is highly subjective and uncertain, a method of quantifying ceramic color characteristics based on the Back Propagation (BP) neural network algorithm is proposed. The semantic difference method and statistical method were used to obtain quantified data from ceramic color perceptual semantic features and were combined with a neural network to study the association between ceramic color features and user perceptual-cognitive evaluation. A BP neural network was used to build a ceramic color perceptual semantic mapping model, using color semantic quantified values as the input layer, color L, A, and B component values as the output layer, and model training to predict the sample. The output color L, A, and B components are used as the input layer and the color scheme was designed. The above method can effectively solve the mapping problem between the appearance characteristics of ceramic color and perceptual semantics and provide a decision basis for ceramic product color design. The case application of color design of daily-use ceramic products was conducted to verify the effectiveness and feasibility of the quantitative research method of ceramic color imagery.

## 1. Introduction

Color is an important appearance feature and aesthetic component of ceramic products, and is a design language that quickly stimulates the user’s emotional experience. Users obtain information about various types of enamel colors through visual senses and process them into imagery descriptions and expressions based on perceptual-cognitive mechanisms [1,2]. Usually, people’s perceptual evaluation of color by visual sense is subjective and uncertain. First, the user’s perception of product color is processed and judged through their aesthetics and experience, which is usually limited by the user’s knowledge level and cultural background. Second, there is no standard to illustrate the intrinsic correlation between the characteristic value of color and semantic degree. There are risks and uncertainties when relying only on the user’s perceptual cognition. The elemental characteristics of the user’s perceptual-cognitive information about ceramic colors are transformed into a scientific and rational data quantification model and applied to the design tools of enamel colors, which is the direction that ceramic product color designers and related users have been focusing on.

In recent years, a large number of results and theories have emerged in the fields of color perceptual description language and color computation quantitative language research at home and abroad, and they are widely used in various fields. For example, Su et al. [3] explored the coupling characteristics of the automotive color-material surface treatment process using latent semantic analysis and summarized the body-color design law based on the high and low modulation of sensory stimuli. Wu et al. [4] established a positive perceptual engineering model for product color design by extracting perceptual imagery information from target products and color design samples and clarified the mapping relationship between perceptual imagery and color samples. Li et al. [5] addressed the hierarchical perception of vision to guide human attentional capture and visual behavior. They proposed using the difference of mutual interference reaction time between two colors to judge their attentional capture degree by correlation analysis of visual perception hierarchy and attentional capture degree based on color system and attentional capture theory. Hsiao et al. [6] proposed a product aesthetics metric based on the color coordinate system and validated the practicality of the method through case studies. Patrick et al. [7] used a Bayesian hierarchical polynomial logit model to model the color preference of target users’ handbags and summarized the design suggestions for product brand color planning.

In recent years, research on human perception of color and color semantics has focused on color harmony, color preference, and color matching, etc. Hard and Sivik [8] proposed a theoretical model of the elements of color harmony; Nemcsics [9] studied the laws of harmony concerning color and proposed a more systematic explanation method; Allesch [10] argued that for single-color and multi-color matchingthere are differences in human preference for monochromatic and multicolor schemes, and such differences exist not only among individuals but also in the relationship between monochromatic and multicolor pairings; color relationships with high luminance and high purity produce strong stimulation for human senses and are more likely to gain people’s preference [11]; Ou et al. [12] established a three-dimensional color semantic model based on color semantics and visual attributes. The current application in own cognition research mainly focuses on descriptive language, which largely excludes direct analysis of perceptual information. However, there are actually many factors that can influence human perception, and by conducting some psychological and physiological experiments, corresponding general rules can be established as a way to obtain more credible perceptual evaluations. Douglas et al. [13] used a transformation of color space very similar to human color perception to measure color, and found that semantically similar words are similar in color distribution, providing a semantic based on metaphorical perception. By proposing a new approach to interpretation, these works provide some support for color perception research.

In addition, in the field of ceramic color design and product color matching technology, many research scholars have conducted explorations around color perceptual cognition and color matching, color detection, data model optimization, and other related issues. For example, Dong et al. [14] proposed a comprehensive color difference method based on the probability density of gray values to control the system of ceramic mosaic paving and produce paving color patterns with different artistic effects. Zhou [15] studied the aesthetic characteristics of the color glaze of ceramic objects from different decorative techniques, integrated decorative concepts to explore the inner meaning of ceramics, and analyzed the aesthetic characteristics of ceramics and the development of artistic creation from the perspective of artistic aesthetic. Zhao et al. [16] studied the aesthetic symbols of ceramic vessels from the color theory of semiotics, analyzing the semantic structure and semantics of color symbols. Wang [17] of Jiangnan University drew on the concept of product color imagery and combined it with modern system design theory to conduct a scientific analysis of color and culture with Jun porcelain as the research object. Wang established a color classification system for Jun porcelain, proposed an imagery scale evaluation method for its color, and verified the feasibility of color semantics and imagery design theory in the field of ceramics. Shen [18] uses semantics and semiotics to sort out Chinese ceramic decorative colors and explores the semantic laws of porcelain glaze colors.

These studies have contributed to ceramic color design to a certain extent. However, there is still a certain lack of design research concerning quantifying the semantic characteristics of ceramic product color. The following deficiencies still exist in the current research on the color level of ceramic products.

(1) The acquisition of color semantics is not precise and difficult to elaborate on. Due to the subjectivity, vagueness, and inaccuracy of users’ cognition, there are ambiguities and one-sidedness in the acquisition. Usually, color theory is based on perceptual cognition and relies only on descriptive semantics, so the user sums up the color law as general and vague, lacking clear numerical representation. At the same time in the ceramic color scheme generation process, the color area ratio, the relationship between the location of the color, and many other factors will also lead to a certain degree of difficulty in ceramic color design.
(2) The existing research has mainly studied the decorative, cultural, and symbolic aspects of ceramic product colors, and has not yet considered the quantification of ceramic color semantic features and personalized recommendations. The different characteristic values of ceramic colors affect the user’s influence, and it is imprecise to determine the color design scheme only through the user’s perceptual cognition. There is a lack of logical operation on the perceptual cognition of ceramic colors, limited systematic exploration of the mapping relationship between color characteristics and perceptual semantics, and no set of design methods for ceramic colors has been formed.

This paper proposes a method to quantify the semantic features of ceramic colors based on a Back Propagation (BP) neural network algorithm. We used optical experimental instruments to collect sample color data combined with perceptual engineering to complete the quantification and analysis of ceramic color characteristics. We applied a BP neural network to establish a data model describing the perceptual-cognitive characteristics of ceramic color to digitally represent color cognition and to solve the problem of mapping between ceramic color appearance characteristics and perceptual semantics. With this model as the basis to build a ceramic color design decision system, we wanted to grasp the inner law of ceramic color and semantics more accurately in product color design, to achieve personalized recommendations for ceramic product color design.

## 2. The Mapping Process of Perceptual Semantic Features of Ceramic Glaze Color

Color information is an abstract cognition of color, and there is a complex psychological reaction process between it and color semantics. Ceramic color perceptual-semantic feature mapping is a way for researchers to simulate the correspondence between color and emotional semantics by establishing a mathematical model to present the complex relationship between color and sensation digitally. By quantifying the mapping relationship between color semantics with the help of computer-aided design, a large amount of ceramic color data can be processed and provide a new approach to enamel color design. Spectrophotometer, also known as a spectrometer, is a scientific instrument that breaks down light with complex composition into spectral lines. The wavelength of the measured light is controlled in the visible region between 380 nm and 780 nm and in the wavelength range of 200–380 nm in the ultraviolet region. Different light sources mean different emission spectra, and different light source instruments can be selected according to the specific requirements of the experiment.

**Figure 1:**
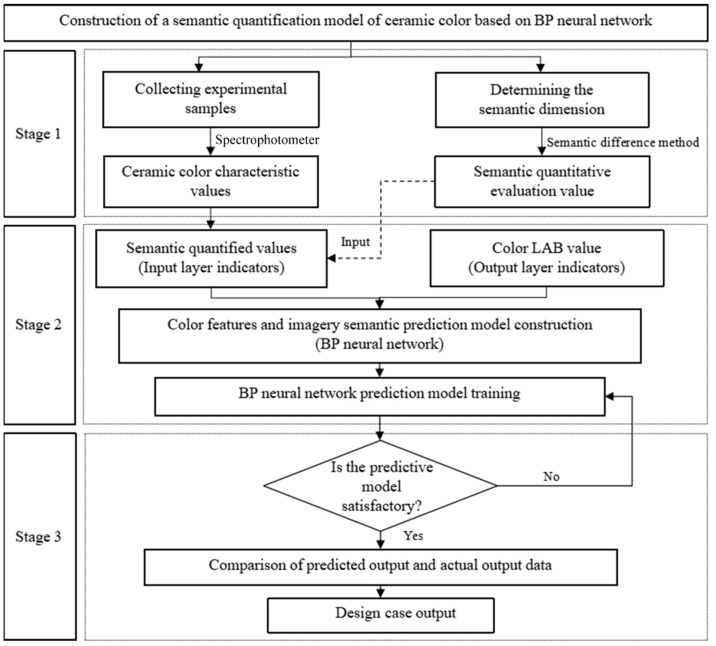
Flow chart of the study.

To accurately describe the characteristic law of ceramic color perceptual semantic cognition, this paper collected sample color data indicators by spectrophotometer. We also used the semantic difference method to obtain the user’s semantic evaluation of color. A BP neural network was used to establish the mapping relationship between the experimental sample and the subject’s semantic evaluation. The specific construction process of a quantitative model for ceramic product color semantic features is shown in Figure 1. The research steps are as follows:

(1) Quantification experiments of ceramic color characteristics and perceptual semantics

Firstly, many experimental samples were collected from different colors of enamel. The color indexes of the samples were collected by using optical instruments, and the relevant data from the experimental samples were obtained by using questionnaires, cluster analysis, and semantic difference methods. The subjects’ perceptual semantic evaluation values of the colors of different experimental samples were recorded. Quantitative processing was then performed by using statistical software for the prerequisite data of the subsequent analysis.

(2) Construction of a quantitative model of ceramic color perceptual characteristics based on BP neural network

By building a BP neural network to process and analyze ceramic sample color indicators and enamel color semantic quantification values, a mapping association model between ceramic color feature values and user perceptual-cognitive evaluation was established. This can be used for the construction of subsequent ceramic product color design systems through model training and prediction.

(3) Ceramic color design case study

After the mapping model was built, the initial experimental data were pre-processed and used as the input parameters of the neural network, and the model was trained to observe the predicted color values and the actual output for comparison. The model calculates the color difference value and the specified error value. These can provide design decisions for the subsequent color scheme of ceramic products and realize the user’s color choice for products with the help of the prototype ceramic color design application system developed later.

## 3. Quantitative Analysis of Ceramic Color Characteristics and Perceptual Semantics

### 3.1. Collection of Enamel Colors and Determination of Semantic Dimensions

Although there is a rich variety of ceramic colors, overall they can be divided into single color and multi-color. In this paper, the collected porcelain enamel color test samples are divided into cyan, yellow, black, red, and multicolor according to the hue. A total of 310 ceramic color samples were collected as experimental samples, including 70 cyan, 70 yellow, 50 black, 60 red, and 60 multicolor. The experiment utilized a spectrophotometer (model X-Rite Pantone Ci7600 benchtop) to collect color data for each ceramic sample, and the *L*, *A*, and *B* values (consisting of the three elements of luminance (*L*) and related color (*A*) and (*B*)) were determined and analyzed for all ceramic samples. Specific test parameters are shown in Table 1 below.

The *L*, *A*, and *B* values of the samples were measured and collated. LAB is a kind of color space, which was built on the basis of the International Color measurement Standards established by the International Lighting Commission (CIE) in 1931. It was revised and named CIELAB in 1976. It is a device-independent color system, and a color system based on physiological properties to describe human visual sensing in a digital way. All the colors visible to the human eye can be displayed through the LAB model, which makes up for the uneven color distribution of the two color modes of RGB and CMYK. The *L*, *A*, and *B* values can macroscopically present the ceramic surface color. *L* represents the brightness index, expressed in black and white. The *L* values range from 0 to 100, *L* = 50, equivalent to 50% of the black. *A* represents the red and green values. *A* positive and larger value means more red, and a negative and smaller value means more green. *B* represents the yellow and blue values. *B* takes a positive value, and the larger the value means more yellow, and the smaller the value, the more blue. Through the characterization of *L*, *A*, and *B* values, small differences in color of each sample can be found. After determining the experimental samples of porcelain enamel color, it is necessary to find the color semantics according to the color factors that match the ceramic products. In this paper, referring to the research results of the literature [19], we use the cluster analysis method to build the cluster relationship, and then after objective evaluation and statistical screening, the semantic word pairs of porcelain enamel color are finally determined in five typical dimensions: {cold-warm}, {hard-soft}, {dark-light}, {artificial-natural}, and {quiet-bright}.

### 3.2. Quantification Method and Standard Development of Color Semantics for Porcelain Enamel

**Figure 2:**
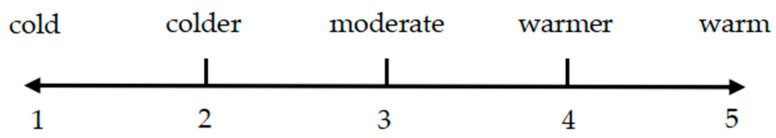
Quantitative grading of the cold-warm semantic dimensions.

To obtain more objective and reliable color perceptual data of ceramic products, this paper adopts the semantic difference method proposed by Osgood [20]. This method aims to quantify the perceptual semantics and establishes a 5-point psychological scale for five dimensions of semantic words, with the range of values taken for each dimensional semantic value set to [1,5]. The values taken represent meanings for semantic levels, with 1 and 5 denoting the ends of the two-color semantic dimensional intervals. The evaluation values given by the testers indicate the degree to which this color object lies at the semantic words at both ends of this semantic interval [21]. For example, “cold-warm” is quantified into five levels, meaning “cold–colder–moderate–warmer–warm”. All five dimensions of semantics are quantified in the same way, where the semantic scale of cold and warm dimensions is shown in Figure 2. If a rating of 5 is given, it means that the emotional imagery conforms to the highest degree of warmth.

The experimental data in this paper were obtained in the form of a questionnaire, and the testers were asked to give their intuitive perception values within the specified range based on the above slag enamel color test samples. To achieve a numerical mapping relationship between the color object and its color semantic information, the mean values from the semantic evaluation of each color’s dimensions were taken. Equation (1) is the formula for calculating the semantic quantification value of each color in each semantic dimension.

(1) $$X_{i}^{j}=\frac{\sum_{q=1}^{n}x_{iq}^{j}}{n}$$

where i is the color sample (0<i<310), j is the perceptual semantic vocabulary dimension (0<j<5). q is the first q subject tested. n is the total number of validly returned questionnaires (0<n<50). $x_{iq}^{1}$ is the rating value given by the first q tester for the first i color in the semantic dimension j. ${Χ}_{i}^{j}$ is the quantified value of color semantics for the first i color sample in the semantic dimension j.

### 3.3. Quantitative Data Acquisition of Perceptual Color Semantic Features of Porcelain Enamel

The collected porcelain enamel sample colors were recorded to quantify their evaluation data under the different semantic dimensions and to provide initial data for the subsequent establishment of the ceramic perceptual color semantic quantification model.

The purpose of this paper is to analyze the user’s psychological experience and perception of ceramic color. The respondents should have certain knowledge and understanding of color appearance, and those who have color weakness, color blindness, or color insensitivities were not included in the survey to ensure that the data were more accurate [22]. The participants in this experiment were all citizens of Chinese nationality and were recruited from the community. In total, 50 participants (25 male and 25 female), with an average age of 22–45 years, were required to be in good health and none reported current or recent (past 12 months) eye diseases or injuries. We excluded those with color defects, color blindness, or color insensitivity from the survey because these defects could affect the accuracy of participants’ color perception and thus the accuracy of the experimental data. Our exclusion criteria relied on participants’ judgment of their own health status and on participants providing written consent prior to the experiment in accordance with procedures approved by the Institutional Review Board of Shaanxi University of Science and Technology.

Ceramic products are loved by many users, and these users include different age groups, different genders, different educational levels, and other aspects of the population. To ensure the universality and reliability of the questionnaire, the participants of the survey were ceramic design students, teachers, and ceramic enterprise designers. A total of 50 test subjects were selected, of which 25 were male and 25 were female. There were 20 ceramic design students, 20 ceramic designers, and 10 ceramic design teachers. The participants were provided with sample pictures of ceramic colors to give their intuitive psychological perception evaluation values, and the evaluation values of all subjects for each semantic dimension were averaged using Equation (1) as the quantitative value of ceramic colors under that dimension. Product color designs are often multi-color with the establishment of color schemes, and a single color often cannot meet the user’s personalized and refined aesthetic needs; therefore, the integration of two colors will produce different visual effects and psychological feelings, and people’s emotional color for a dual color scheme is much more complex than the psychological feelings of a single-color color, so the mapping of a dual color scheme color and perceptual semantics is worth studying. Therefore, the mapping of color and perceptual semantics is worth studying. We collected the 310 different ceramic color samples needed for this paper, and all color samples were first randomly combined two by two, with a total of 47,895 two-color matching schemes. Then, 500 groups of color samples were randomly selected for the survey experiment, as shown in Table 2. The ceramic color samples were numbered sequentially from 1 to 500. The serial numbers corresponded to color and semantic quantification values to facilitate the recording of data and subsequent data processing.

The data obtained from this test were processed according to the above quantification method, and the semantics of each color were calculated statistically using Equation (1). The semantic quantification values given by the test subjects were averaged as the semantic quantification results, as shown in Table 3 for the processed semantic quantification data.

## 4. Construction of a BP Neural Network-Based Quantitative Model for the Perceptual Characteristics of Ceramic Color

In the previous phase of this paper, relevant data from test samples were obtained in the form of questionnaires, but often the results from questionnaires are not extensive, so the obtained color and perceptual semantics do not form a specific pattern between them. The study showed that the experimental results are subject to human influence, and factors such as participants’ age, background, aesthetic preference, and quantity will have uncertain effects on the experimental results. Age is also considered to be an important variable that affects perception and cognition over time. Usually the average age is 40 years when color perception has changed, and when reaching the age of 75 years, the expressiveness of color perception decreases more easily, causing a weakening of color perception changes or loss of color discrimination [23]. A study also showed that the age of the participants does not change the perception of positive emotions, but changes negative emotions [24]. Aesthetic preference is a work that belongs to product aesthetics. Sondereger and Sauer [25] analyzed product aesthetics and determined its influence on perceived usability, and color is an important aesthetic factor that can influence the relationship between humans and products. In addition, the quantitative factor is when the questionnaire experiment reaches a certain number of criteria to support the credibility and validity of the questionnaire analysis. In this paper, we invited students, teachers, and corporate designers, whose aesthetic preferences and experiences are different, so as to ensure the diversity and comprehensiveness of the sample; at the same time, we also made sure to reach a certain degree in terms of the number to ensure the questionnaire’s universality and reliability.

Due to the influence of differences in the number of subjects, their age, aesthetic preferences, and the relationship between color and semantics studied in this paper will be a complex existence and no longer a simple linear relationship. The neural network, with its powerful functions of self-learning, self-adaptive, nonlinear, and distributed parallel processing, is widely used in many fields such as optimal combination and prediction. Therefore, this paper investigates the complex, nonlinear mapping relationship between ceramic color and semantics with the help of a BP neural network model.

### 4.1. The Basic Working Process of a BP Neural Network

**Figure 3:**
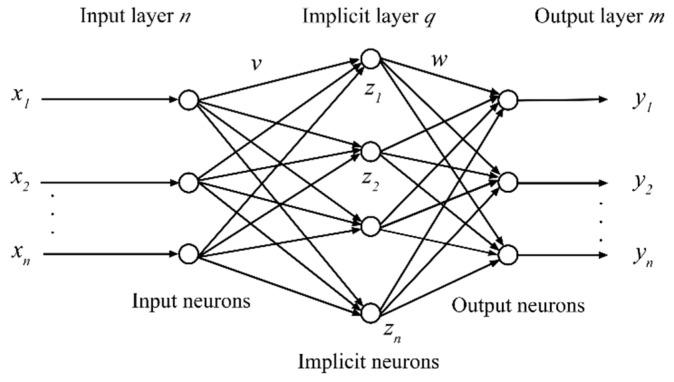
Structure of three-layer BP neural network.

Back Propagation neural networks are one of the most widely used models in artificial neural networks. Essentially, they are multilayer feedforward networks that mainly use an error back propagation algorithm to carry out specific work [26]. Figure 3 shows a typical 3-layer topological neural network structure, including an input layer, an intermediate hidden layer, and an output layer.

The basic working process of a BP neural network is: given a sample set, first obtain the feature vector of the sample set as the input vector of the network through the forward channel propagation to the hidden layer nodes. Each node is a neuron with a nonlinear activation function. The input vector works with the assistance of the activation function, so that the output point can receive the information sent by the hidden node to derive the specific results of the operation and calculate the network. The minimum mean squared difference value between the actual output and the desired output is used as the objective function, and the weights and thresholds are continuously corrected to minimize the network error [27].

### 4.2. Semantic Model of Ceramic Color Based on BP Neural Network

In this paper, the mapping relationship between ceramic color and perceptual semantics was investigated using BP neural networks, assuming a single hidden layer neural network structure, defined in Figure 3 as follows.

n—the number of neurons in the input layer.

q—the number of neurons in the hidden layer.

m—the number of neurons in the output layer.

$x_{1},x_{2},\cdots ,x_{n}$—five-dimensional semantic quantification values.

$h_{1},h_{2},\cdots ,h_{q}$—the output of the implicit layer.

$y_{1},y_{2},\cdots ,y_{m}$—color *L*, *A*, and *B* component values.

${\hat{y}}_{1},{\hat{y}}_{2},\cdots ,{\hat{y}}_{n}$—the predicted *L*, *A*, and *B* component values of the training sample.

$v_{ij}$—the weight of the input layer cell i to the implied layer cell j.

$w_{jk}$—Connection weights from the implicit layer unit j to the output layer unit k.

The mathematical model of the output of each hidden layer neuron is shown in Equation (2).

(2) $$z_{j}=f\left(\overset{n}{\underset{i=1}{\sum }}v_{ij}x_{i}-\theta_{i}\right),j\in \left\{1,2,\cdots q\right\}$$

where i is the input layer cell, j is the implied layer cell, and $\theta_{i}$ is the threshold value of the input layer.

The output of the output layer node unit is as shown in Equation (3).

(3) $$y_{k}=f\left(\overset{q}{\underset{j=1}{\sum }}w_{jk}z_{j}-\phi_{k}\right),k\in \left\{1,2,\cdots ,m\right\}$$

where $y_{k}$ is the output value of the output layer node cell, $z_{j}$ is the number of j hidden layer nodes, and $\phi_{k}$ is the threshold value of the output layer.

The error between the network output and the predicted output is defined in terms of the mean square error value also known as the objective function expression, as shown in Equation (4).

(4) $$E=\frac{1}{2}\;\overset{m}{\underset{k=1}{\sum }}{\left(y_{k}-{\hat{y}}_{k}\right)}^{2}$$

where Ε is the mean square error, $y_{k}$ is the validation sample network output *L*, *A*, and *B* values, and ${\hat{y}}_{k}$ is the training prediction output *L*, *A*, and *B* values.

When the error does not reach the set target set value, i.e., the network output does not achieve the expected effect, the network model adjusts the correction of the weights according to the error between the actual output and the expected output until the network output error reaches the expected result, then the network training is finished.

### 4.3. BP Neural Network Model Parameter Setting

**Figure 4:**
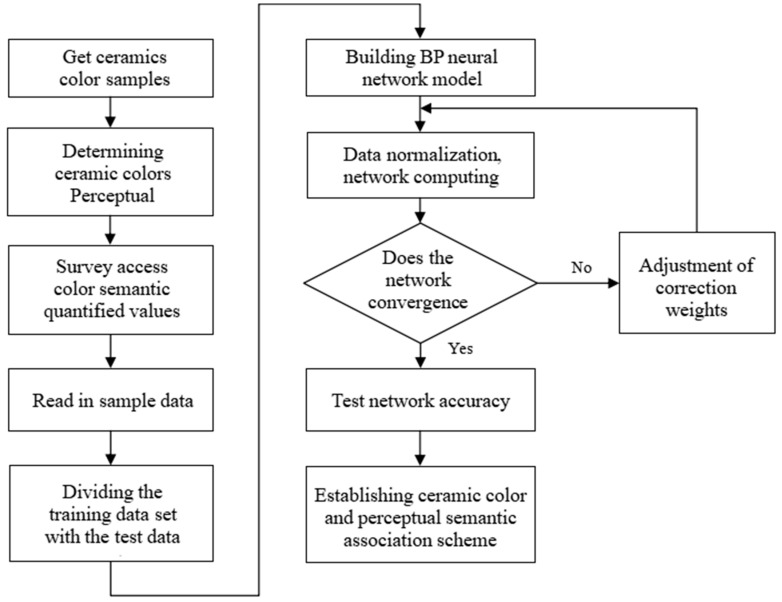
Flow chart of ceramic color semantic mapping model.

The five-dimensional perceptual semantic quantification values described above were selected as the network input variables, and the mapping relationships between the three component values of color *L*, *A*, and *B* and the perceptual semantics were independent of each other. Therefore, the *L* component, *A* component, and *B* component of each color were used as the outputs of three independent neural networks. Figure 4 shows the flowchart of the ceramic color semantic mapping model design, from which the system calculates the nonlinear relationship between ceramic color values and perceptual semantic quantization values. This gives a clear, guiding analysis of the mapping between perceptual color semantics and color values.

In this experiment, 500 groups of samples were randomly selected from the random two-color combination scheme of ceramic color samples of the five color families described in Section 3.3. The semantic quantization values of the five dimensions corresponding to each group of colors were obtained according to the color semantic quantization method described in this paper as the five input values of the neural network. The output values of the three opposing neural networks are the *L* component value, *A* component value, and the *B* component value of each color, respectively. The output layer of the neural network model structure should contain two neurons, which output the *L*, *A*, and *B* values of two colors respectively. The number of neurons in the input and output layers of the neural network established in this paper are 5 and 2, respectively.

#### 4.3.1. Sample Data Pre-Processing

The input variables of the neural network built in this paper are the semantic quantization values of ceramic colors, which take values between [1, 5], and the output variables are the measured *L*-component values, *A*-component values, and *B*-component values, with the value range of *L* being [0, 100] and the value range of both *A* and *B* components being [−127, 128]. To reduce the impact of singular sample data on the performance of the network training results, and to reduce the number of network operations, the input and output parameter data need to be normalized to the same scale [28], so that the data take values in the range between 0 and 1. The normalization method is as in Equation (5).

(5) $$y=\frac{x-x_{\min}}{x_{\max}-x_{\min}}$$

where *x* is the input variable and *y* is the normalized value of the data.

The actual variation range of *x* is [*x*_min_, *x*_max_], which is calculated by the formula transformation so that the variation range of sample parameters is within the interval of [0, 1]. This can effectively reduce the amount of network training computation as well as improve the network training performance.

#### 4.3.2. BP Neural Network Design

This experiment used a three-layer BP neural network model, including the input layer, the intermediate hidden layer, and the output layer. The input layer has five nodes and the output layer contains two nodes. The number of neurons in the intermediate hidden layer is set to n and is adjusted by experiment to finally determine the optimal number of nodes [29].

Choosing the appropriate activation function can make the neural network have a more powerful fitting ability and be more effective in realizing the nonlinear complex mapping between the input and output, an important part of the process of building neural networks [30]. In this paper, we used the most common Sigmoid activation function, whose mathematical expression is (6).

(6) $$f(x)=\frac{1}{1+e^{-x}}$$

**Figure 5:**
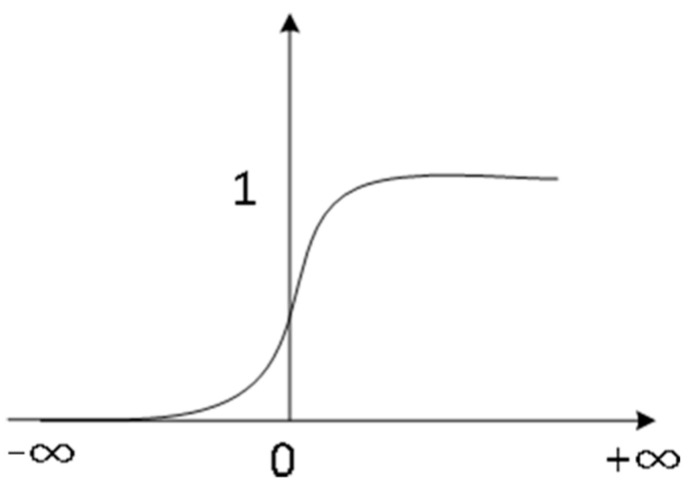
Sigmoid function.

A Sigmoid function is a nonlinear function, commonly used as the output function of the implicit layer. Its value range between [0, 1], that is, the value of (−∞,+∞) number of output mapping to [0, 1] between the mean value of the Sigmoid function is 0.5 [31]. The derivative of the Sigmoid function can be obtained as a function of its own, $f^{\prime }(x)=\frac{e^{-x}}{1+e^{-x}}=f(x)*(1-f(x))$, which will largely reduce the back propagation algorithm to find the error gradient step. The graph of the Sigmoid function is shown in Figure 5.

In this paper, the input layer, and the hidden layer of the neural network both use the Sigmoid function as the transfer function. The application of the Sigmoid function in the neural network has been proven to obtain better results, as it combines the advantages of linear and nonlinear functions and smooth continuous transformation in the middle of the input and output data. Under the action of the Sigmoid function, nonlinear factors are incorporated into the neurons so that all the nonlinear functions can be associated with the neural network, and the output layer can just use the linear transfer function (Purelin) so that the network can output any range of values, as shown in Equation (7).

(7) f(x)=x

#### 4.3.3. BP Neural Network-Based Semantic Mapping Prediction Model

**Figure 6:**
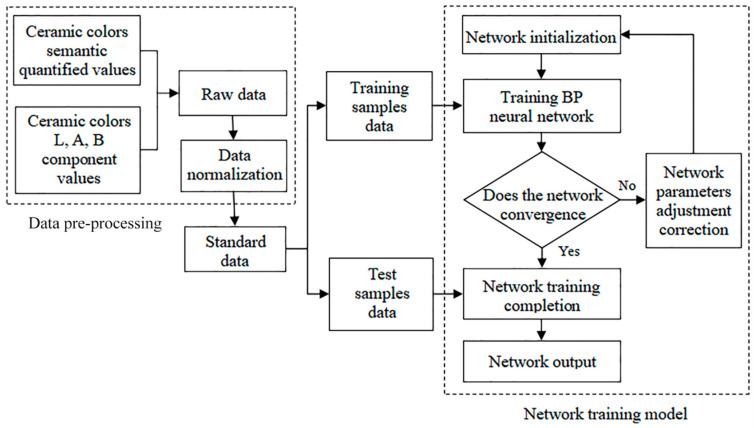
Structure of the semantic mapping model of ceramic color based on BP neural network.

First of all, to obtain the training samples for the 500 groups of color semantic samples collected, the original sample data needed to be normalized to make the data have the same level and facilitate the network operation. The standard sample data set obtained was divided into two groups: the training sample set and the test sample set. A total of 400 groups of data were extracted as training samples and the remaining 100 groups were used as test samples. The test set was used to test the fitting and prediction ability and generalization ability of the trained neural network [32]. Figure 6 shows the structure of the semantic mapping prediction model of ceramic color based on the BP neural network.

After the neural network is trained, it can output the predicted color *L*, *A*, and *B* values under different semantics, and subsequently compare them with the color values of ceramic samples stored in the database to calculate the color error. According to the error result, the color of the sample with the smallest error will be displayed in the system interface as the color value that best matches the current semantics. The mapping model establishes a correspondence between ceramic color and semantics, which can quickly assist ceramic product color designers and related users in the color selection. If users want to adjust the color scheme predicted by the model, they can select a closer color according to the results generated by the network for fine-tuning and then get the final color design scheme.

## 5. Ceramic Color Design Case Study

The initial experimental data obtained in the previous section were pre-processed. That is, the perceptual semantic values of Table 2 were normalized by Equation (5) and used as the input parameter quantity of the neural network, and then the color prediction *L*, *A*, and *B* component values of the test samples were calculated using by Equations (6) and (7). The difference between the predicted output value and the actual output value was calculated, and the reliability of the model was verified after comparison. The corresponding color *L*, *A*, and *B* component values of the actual output were obtained through the inverse normalization of the output values and can be used to guide the design of subsequent color schemes for ceramic products.

### 5.1. Experiments and Results Analysis

**Figure 7:**
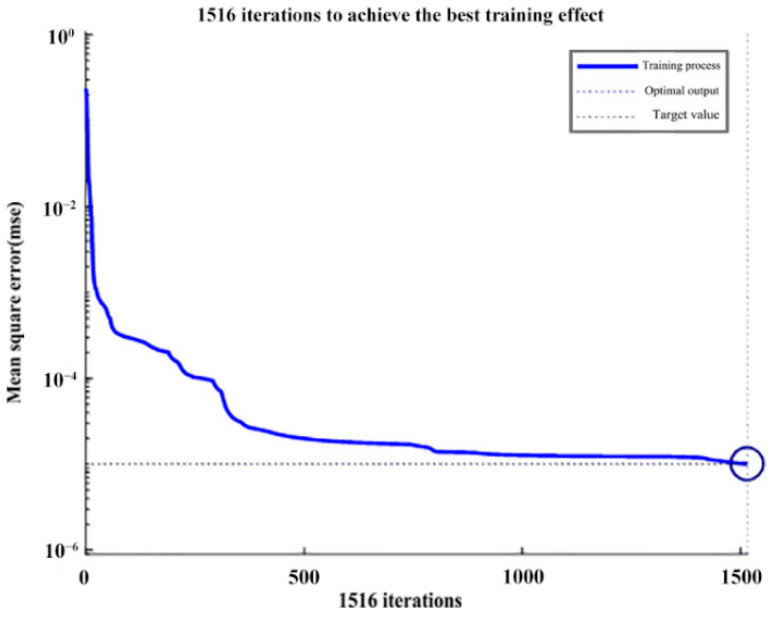
Learning process curve of the network model of complex color perceptual mapping.

This experiment used a MATLAB software neural network toolkit to build a three-layer BP neural network model. Reasonable network parameters were set through continuous experiments, including the number of training times, the learning rate, the number of neurons in the hidden layer, and the learning target function so that the network can learn to train the input sample data [33]. This study built a test 5-N-2 structure of the network model, the mean square MSE as the learning target was set to 1 × 10^−6^, and the learning rate was set to 0.01 by continuous debugging to achieve the best results. This can better balance the contradiction between learning time and learning effect. The maximum number of training was set to 3000, and the network was tested several times. The training was continuously tuned, and the results showed that the number of nodes in the hidden layer N was taken between 25–30 to obtain a better performance of the network structure and the experimental results. Taking the *L*-component value as an example for analysis, the learning process curve of the network model trained with the structure of 5-30-2 is shown in Figure 7.

**Figure 8:**
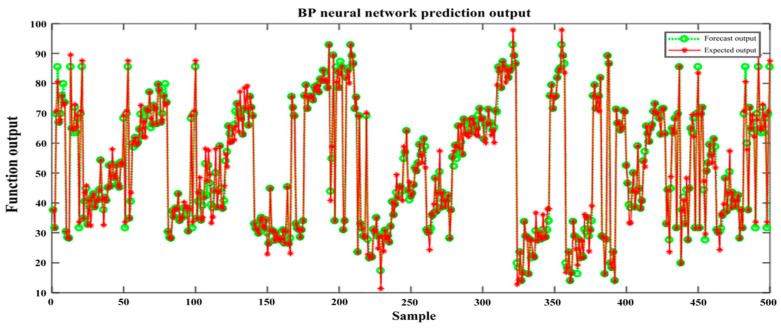
BP neural network simulation results.

With the increasing number of training times, the network is adjusted by continuous learning, and the network mean square error converges until it reaches the preset target value, as seen in the circle in Figure 7. The network learned 1516 times when the target error reached the set learning target value, at which time the BP neural network model was trained. The trained network output took the value range of [0, 1], and the actual output corresponded to the color *L* component value, which takes the value range of [0, 100]. It is necessary to inverse normalize the network output data to get the actual luminosity *L* component value, and the corresponding inverse normalization process is carried out for *A* and *B* components, respectively. The actual output value is compared with the network prediction value to test the network learning performance and accuracy. In this experiment, 100 test set samples without network learning were used to test the generalization and learning ability of the network model [34], as shown in Figure 8. For the first color *L* component value of the predicted output of the BP neural network, it can be seen that the prediction results are good and can predict the output of the nonlinear function between color semantic mappings.

The partial results of the neural network simulation and the actual output values are shown in Table 4, and the errors are calculated for subsequent analysis and processing. Full data can be accessed by clicking this link: https://osf.io/vwz7g/?view_only=3547074bfe94431cad90e11078ecdcf2, accessed on 19 June 2022.

From the network training results, the maximum errors of *L*, *A*, and *B* components can be calculated as Δ*L*1 = 2.43, Δ*L*2 = 1.92, Δ*A*1 = 1.35, Δ*A*2 = 1.94, Δ*B*1 = 2.08, Δ*B*2 = 1.37. In engineering, the difference in color perception between two sample colors is generally evaluated by the color difference Δ*E*. Δ*E* is defined as the total color difference between the two colors, and the color difference formula is widely used in ceramics, plastics, and textiles industries. The expression for calculating the color difference is shown in Equation (8).

(8) $$\Delta E=\sqrt{{\left(\Delta L\right)}^{2}+{\left(\Delta A\right)}^{2}+{\left(\Delta B\right)}^{2}}$$

Using Equation (8), we calculated Δ*E*1 = 3.378, Δ*E*2 = 3.157 for the evaluation criteria of the error. According to the national standard issued by the national requirements for color quality of decorative printed products, the same batch of the same color difference is defined as general products Δ*E* ≤ 5.00~6.00. Fine products require Δ*E* ≤ 4.00~5.00 [35]. By comparison, this BP neural network training can obtain a better learning effect and can generate a color design scheme for the user. Therefore, the BP neural network structure established in this paper has certain reliability and applicability for ceramic color perceptual element mapping and is desirable for ceramic product color scheme design.

### 5.2. Ceramic Product Color Design Scheme

In view of the previous simulation results and analysis in the MATLAB environment, the feasibility of the algorithm proposed in this paper is verified. This paper uses the C++ programming language for the programming implementation of the algorithm. The software system was developed based on the Microsoft Foundation Classes (MFC) class library and the Microsoft Visual Studio 2010 (VS2010) development platform.

**Figure 9:**
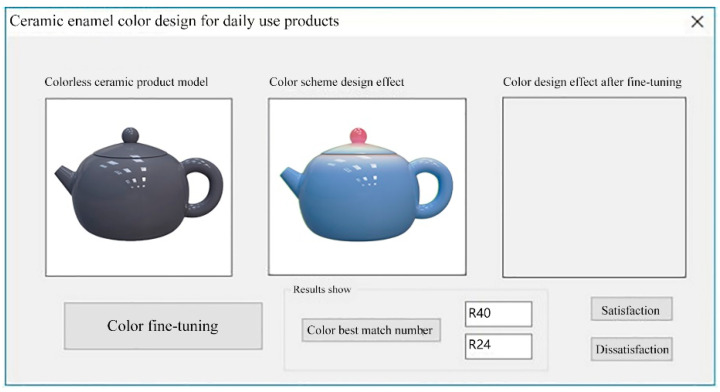
Ceramic daily use product re-color effect.

The user enters the required scores under the five semantic dimensions in the semantic parameter setting area, sets the appropriate network parameters in the network training module, conducts model training, and gets the color values predicted by the network after completion. The color scheme design for ceramic products is based on the color values obtained from the neural network training, and the final effect is displayed in the interface shown in Figure 9. The left side shows the model of ceramic products without color, and the middle will show the effect of the neural network-generated color scheme design. In addition, the result value output by the system will be calculated with the color value of ceramic color samples stored in the database to calculate its error. The sample with the smallest error will be used as the most matching slag enamel sample, and its corresponding number will be displayed in the interface result display area, meaning that the correspondence between ceramic color and semantics is established.

**Figure 10:**
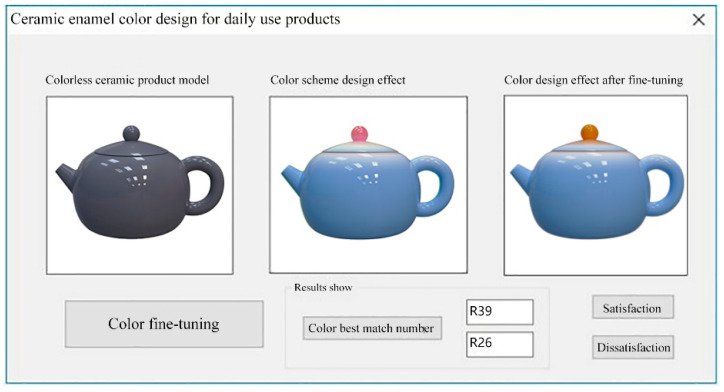
Effect of re-coloration of ceramic daily products after fine adjustment.

If the result of the neural network training color scheme is consistent with the user’s expected results, they click on the “Satisfy” button. If the user needs to adjust the system-generated color scheme, they can select the “color fine-tuning” button. The pop-up color plate, using the network-generated color results as the recommended color scheme, selects the closest to the ideal color scheme for fine-tuning, and a satisfactory color scheme is presented. The new enamel color scheme is compared with the sample number in the ceramic color classification table, and the interface shows the corresponding ceramic color number, as shown in Figure 10.

**Figure 11:**
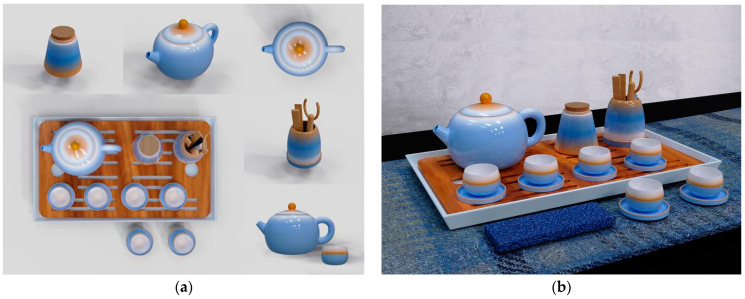
Tea set series product design scheme. (**a**) Single product model effect; (**b**) The overall effect of the series.

In this paper, we take the tea set series of daily-use ceramic products as an example for program design (see Figure 11). The final rendering effect of each ceramic without a color model is shown in Figure 11a, and the overall color matching effect of the series is shown in Figure 11b.

## 6. Conclusions

A neural network-based quantitative prediction model and design system for ceramic color semantics were investigated and the following conclusions were drawn.

(1) A preliminary system structure of the ceramic color feature quantification method was established. Based on the theory of perceptual engineering, the research on the theory, method, and viewpoint of color perceptual elements characteristics was explored in depth, along with the aspects of quantification of user perceptual elements and color design of porcelain enamel. Through the quantification of ceramic color elements, certain theoretical methods are provided for the design application of ceramic colors in products.
(2) The quantification process of ceramic color characteristics was studied. The ceramic color sample data and images were collected, organized, and analyzed. A ceramic color design model based on a BP neural network algorithm was proposed, and a mapping model between ceramic color samples and perceptual semantics was established to transform perceptual-cognitive elements into a computationally quantifiable model, providing a basis for decision-making in ceramic product color design.
(3) A prototype system for ceramic color design was constructed. Based on the evaluation and analysis data of the ceramic perceptual color element feature mapping relationship model, the prototype system of ceramic color feature quantification and design application was created by using the C++ language and MFC framework. At the same time, the main functional application modules of the prototype system and the demonstration process of the application cases are given.

Combined with the actual application effect of the system, the feasibility and effectiveness of the theoretical system and method proposed in this paper are further verified.

## Figures and Tables

**Table 1:** Photometric test parameters.

Parameter Name	Parameter Values
Reatability (white film)	0.03RMS△ECIELAB *
Inter-instrument table difference	mean 0.15△ECIELAB
Light source	Pulsed xenon, D65 calibration
UV filter	400 nm
Spectral range	360–750 nm
Wavelength Accuracy	<0.1 nm
Wavelength Precision	<0.05 nm
Wavelength interval	10 nm
Bandpass filter	10 nm
Photometric range	0–200%
Photometric resolution	0.01%
Measurement cycle time	≈2.5 s
Color Preview	Video and color sample door preview
Reflective Aperture	25 mm
Full transmittance aperture	22 mm
Direct transmission	22 mm

△E represents the difference value between the measured CIELAB values of the two groups. * represents high precision mode.

**Table 2:** Ceramic glaze sample color information table.

Sample	*L*		*A*		*B*		Color
1	*L*11	29.94	*A*11	−0.26	*B*11	−2.2	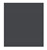
	*L*12	63.16	*A*12	−7.88	*B*12	4.07	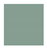
2	*L*21	85.51	*A*21	−1.96	*B*21	1.64	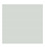
	*L*22	63.46	*A*22	−11.03	*B*22	8.65	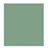
3	*L*31	29.86	*A*31	13.42	*B*31	21.18	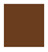
	*L*32	59.49	*A*32	2.37	*B*32	18.53	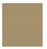
4	*L*41	78.76	*A*41	0.49	*B*41	10.75	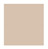
	*L*42	92.86	*A*42	1.8	*B*42	6.44	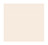
5	*L*51	64.1	*A*51	5.27	*B*51	29.91	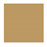
	*L*52	71.42	*A*52	−5.32	*B*52	−4.6	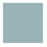
6	*L*61	79.65	*A*61	−14.09	*B*61	−7.1	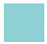
	*L*62	49.18	*A*62	11.75	*B*62	11.01	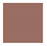
7	*L*71	34.23	*A*71	6.49	*B*71	0.71	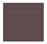
	*L*72	35.75	*A*72	−8.68	*B*72	7.83	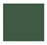
8	*L*81	67.15	*A*81	−11.09	*B*81	7.58	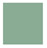
	*L*82	64.1	*A*82	5.27	*B*82	29.91	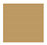
…	…	…	…	…	…	…	…
500	*L*1001	84.53	*A*1001	1.07	*B*1001	3.77	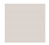
	*L*1002	75.78	*A*1002	−11.62	*B*1002	−1.48	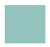

Note: Data from spectrophotometric instruments.

**Table 3:** Data table of ceramic color semantic quantification samples.

Sample	Cold-Warm	Hard-Soft	Dark-Bright	Artificial-Natural	Quiet-Lively
1	1.9	3.58	1.48	2.86	1.72
2	1.02	1.12	0.66	4.44	1.08
3	1.78	1.22	1.14	3.64	1.68
4	3.94	1.01	1.02	4.64	1.11
5	1.44	1.28	1.3	3.74	1.72
6	1.56	1.4	1.8	3.2	1.84
7	4.62	4.29	1.82	2.92	2
8	1.92	1.9	1.48	2.8	1.92
…	…	…	…	…	…
500	1.02	1.22	1.22	4.16	1.05

Note: Data from questionnaire research results.

**Table 4:** Simulation results of the semantic mapping model of ceramic two-color matching perceptual based on BP neural network.

**Sample**	**Test Sample *L*, *A*, *B* Actual Values**					
	***L*1**	***L*2**	***A*1**	***A*2**	***B*1**	***B*2**
1	71.52	80.82	−0.78	1.76	2.44	26.51
2	94.95	40.98	2.37	−6.69	18.53	13.3
3	59.49	40.79	−4.9	−8.46	19.37	−13.6
4	59.24	62.28	−14.71	−0.69	−7.72	0.02
5	66.43	35.91	15.47	1.07	15.66	3.77
6	46.54	84.53	0.43	0.85	−1.5	6.79
7	85.4	93.06	−9.88	−4.59	−0.22	17.2
8	77.78	56.26	−8.71	−4.24	10.26	9.96
9	63.17	57.12	0.41	−7.02	−0.13	−10.1
…	…	…	…	…	…	…
100	52.5	69.11	4.21	1.79	−2.85	16.79
**Sample**	**Neural Network Simulation Predicted Values**					
	***L*1′**	***L*2′**	***A*1′**	***A*2′**	***B*1′**	***B*2′**
1	73.91	80.27	−0.12	2.04	3.02	24.43
2	93.27	42	3.09	−6.02	19.12	12.67
3	60.83	41.67	−5.24	−9.02	20.67	−12.68
4	61.47	64.1	−16.06	−1.2	−8.12	−0.13
5	65.23	36.12	16.02	1.78	16.23	4.03
6	45.53	83.12	0.91	1.21	−1.89	7.23
7	84.54	92.25	−10.2	−5.32	−0.45	16.22
8	76.53	55.34	−7.43	−5.68	11.2	10.8
9	64.67	57.98	1.21	−8.32	−1.8	−11.23
…	…	…	…	…	…	…
100	51.71	69.19	5.43	2.45	−3.02	17.1
**Sample**	**Error**					
	**Δ*L*1**	**Δ*L*2**	**Δ*A*1**	**Δ*A*2**	**Δ*B*1**	**Δ*B*2**
1	2.39	0.55	0.66	0.28	0.58	2.08
2	1.68	1.02	0.72	0.67	0.59	0.63
3	1.34	0.88	0.34	0.56	1.3	0.92
4	2.23	1.92	1.35	0.51	0.4	0.15
5	1.2	0.21	0.55	0.71	0.57	0.26
6	1.01	1.41	0.48	0.36	0.39	0.44
7	0.86	0.81	0.32	0.73	0.23	0.98
8	1.25	0.92	1.28	1.54	0.94	0.84
9	1.5	0.86	0.8	1.3	1.67	1.13
…	…	…	…	…	…	…
100	0.79	0.08	1.22	0.66	0.17	0.31

## Data Availability

The data used to support the findings of this study are included in the paper.
